# Cysteine-specific ^89^Zr-labeled anti-CD25 IgG allows immuno-PET imaging of interleukin-2 receptor-α on T cell lymphomas

**DOI:** 10.3389/fimmu.2022.1017132

**Published:** 2022-12-15

**Authors:** Jin Hee Lee, Kyung-Ho Jung, Mina Kim, Kyung-Han Lee

**Affiliations:** ^1^ Department of Nuclear Medicine, Samsung Medical Center, Sungkyunkwan University School of Medicine, Seoul, Republic of Korea; ^2^ Samsung Advanced Institute for Health Sciences & Technology, Sungkyunkwan University School of Medicine, Seoul, Republic of Korea

**Keywords:** CD25, antibody, immuno-PET, lymphoma, regulatory T cells, ^89^Zr

## Abstract

**Introduction:**

Positron emission tomography (PET) using radiolabeled Abs as imaging tracer is called immuno-PET. Immuno-PET can verify therapeutic Ab delivery and can noninvasively quantify global levels of target expression in tumors of living subjects. The interleukin-2 receptor α chain (IL-2Rα; CD25) is a promising target for immune therapy and radioimmunotherapy of lymphomas. Immuno-PET could facilitate this approach by visualizing CD25 expression *in vivo*.

**Methods:**

We prepared ^89^Zr-anti-CD25 IgG specifically labeled to sulfhydryl moieties by maleimide-deferoxamine conjugation.

**Results and Discussion:**

CD25(+) SUDHL1 human T-cell lymphoma cells showed high anti-human ^89^Zr-CD25 IgG binding that reached 32-fold of that of CD25(-) human lymphoma cells and was completely blocked by excess unlabeled Ab. In SUDHL1 tumor-bearing nude mice, pharmacokinetic studies demonstrated exponential reductions of whole blood and plasma activity following intravenous ^89^Zr-anti-CD25 IgG injection, with half-lives of 26.0 and 23.3 h, respectively. SUDHL1 tumor uptake of ^89^Zr-CD25 IgG was lower per weight in larger tumors, but blood activity did not correlate with tumor size or blood level of human CD25, indicating minimal influence by circulating soluble CD25 protein secreted from the lymphoma cells. ^89^Zr-CD25 IgG PET allowed high-contrast SUDHL1 lymphoma visualization at five days. Biodistribution studies confirmed high tumor ^89^Zr-CD25 IgG uptake (8.7 ± 0.9%ID/g) that was greater than blood (5.2 ± 1.6%ID/g) and organ uptakes (0.7 to 3.5%ID/g). Tumor CD25-specific targeting was confirmed by suppression of tumor uptake to 4.3 ± 0.2%ID by excess unlabeled CD25 IgG, as well as by low tumor uptake of ^89^Zr-labeled IgG2a isotype control Ab (3.6 ± 0.9%ID). Unlike CD25(+) lymphocytes from mouse thymus that showed specific uptake of anti-mouse ^89^Zr-CD25 IgG, EL4 mouse lymphoma cells had low CD25 expression and showed low uptake. In immunocompetent mice bearing EL4 tumors, anti-mouse ^89^Zr-CD25 IgG displayed low uptakes in normal organs as well as in the tumor. Furthermore, the biodistribution was not influenced by Ab blocking, indicating that specific uptake in nontumor tissues was minimal. ^89^Zr-CD25 IgG immuno-PET may thus be useful for imaging of T-cell lymphomas and noninvasive assessment of CD25 expression on target cells *in vivo*.

## Background

The most common hematologic malignancy in adults is lymphoma. For B cell lymphomas, incorporation of a monoclonal antibody (Ab) called rituximab that targets CD20 into the regimen has markedly improved patient survival (1). In comparison, the prognosis of T cell lymphomas remains poor from frequent treatment resistance and disease relapse (2). As such, newer targets for Ab agents need to be identified to improve the prognosis of relapsed or refractory T cell lymphomas (3).

A promising target that is abundantly expressed on T cell lymphomas is the α chain of the interleukin-2 receptor (IL-2Rα), also known as CD25. This receptor, first cloned from adult T cell leukemia lymphoma, is associated with greater lymphoma aggressiveness and worse patient outcomes (4, 5). The observation that T lymphomas have high expression of this receptor whereas normal cells (other than subsects of regulatory and activated T lymphocytes) do not show its expression has led to the development of novel CD25-directed treatment strategies (6). Hence, clinical trials are ongoing with anti-CD25 Abs conjugated with toxins or particle-emitting radioisotopes. Cytotoxin-conjugated anti-CD25 Ab can kill lymphoma cells and induce higher remission rates for CD25(+) compared to CD25 (–) lymphomas (7). Furthermore, β‐emitting radionuclide-labeled anti‐CD25 Abs have shown promising preclinical efficacy in models of lymphoma and leukemia (8–10). In clinical studies, an early trial of ^131^I-labeled anti-CD25 Ab successfully induced responses patients with refractory CD25(+) lymphoma (11). More recently, ^90^Y-labeled daclizumab (a humanized Ab against CD25) is demonstrating therapeutic promise (12–14).

Furthermore, CD25 is also expressed on activated regulatory T cells (Tregs) that can contribute to the tumor’s ability to evade immune detection (15). Because the presence of large numbers of Tregs infiltrating the tumor is associated with poor prognosis (16), these cells are attracting attention as another target for treatment with Abs against CD25.

The success of CD25-targeted Ab therapeutics can be facilitated by newer diagnostic techniques that can improve patient selection and response prediction. Treatment response, in turn, is determined by the presence of sufficient target antigen expression. Although immunohistochemistry examination of biopsied specimens is the current standard for assessing antigen expression, the accuracy of this method is hindered by tumor heterogeneity and sampling bias (17, 18). Positron emission tomography (PET) can overcome the current limitations of sampling error, invasiveness, and difficulty of serial examination (19). Immuno-PET that exploits radiolabeled Abs as imaging probes can confirm delivery of Ab therapeutics to the tumors and can further assess global target amount without the influence by tumor heterogeneity. PET imaging using therapeutic Abs conjugated with positron emitters is particularly well-poised to help optimize cancer radioimmunotherapy. Indeed, immuno-PET has been demonstrated to detect radioimmunotherapy targets on lymphoma cells with high sensitivity and resolution (20–22). As such, immuno-PET could have a substantial positive impact on the advancement of Ab therapeutics for lymphomas and may provide an *in vivo* companion diagnostic tool that can contribute to personalized treatments.

We found one recent study related to CD25-targeted immuno-PET that used a ^124^I-labeled Ab for imaging of activated T cells (23). Although ^124^I has a long half-life that allows delayed imaging, it suffers from a major drawback for immuno-PET because it fails to remain in target cells following internalization. After receptor-bound Ab is internalized, free ^124^I is released from the Ab by catabolic dehalogenation and escapes the cells. This has undesirable consequences of reduced target signal and increased unspecific thyroid accumulation (24). In comparison, radiometal chelates have residualizing properties that benefit *in vivo* imaging of Abs that bind to target antigens. This has driven immuno-PET development toward the use of ^89^Zr, a positron emitting radiometal with a 3.3-day half-life suitable for sufficient blood clearance of Abs. Internalized ^89^Zr is trapped inside target cells and provide high resolution and high-contrast PET images over prolonged duration after administration.

For the successful development of immune-PET against CD25, an optimal ^89^Zr conjugation technique is required. Owing to established chemical properties and safety profile, the bifunctional chelating agent desferrioxamine (DFO) is most frequently used for ^89^Zr labeling to Abs (25, 26). A simple conjugation method is to target lysine residues, but this produces an inhomogeneous mixture of Ab-chelators with various substitution ratios, and random reaction with ϵ-amino groups in the antigen binding domain reduces Ab immunoreactivity. Our group established a straightforward technique to link ^89^Zr specifically to hinge-region disulfide bonds of Abs through a mild reduction reaction followed by maleimide-DFO conjugation. ^89^Zr-labeled Abs thus prepared provided high affinity and tumor target- specific binding *in vitro* and high-contrast PET images *in vivo* (27–30).

Another requirement is an appropriate preclinical model that considers properties that could influence tumor uptake of anti-CD25 Ab. Immune deficient mice that lack normal T cells could dissect uptake by human lymphoma cells from uptake by infiltrating Treg cells. As another interesting factor, a previous study suggested that serum IL2-Rα released from large tumors might influence tumor delivery of radiolabeled anti-CD25 Ab fragments (31). Whether ^89^Zr-anti-CD25 Ab activity in the blood is influenced by tumor size or circulating CD25 concentration thus requires evaluation.

In this study, we prepared cysteine site-labeled ^89^Zr-anti-CD25 IgG for specific targeting and PET imaging of human T cell lymphomas in nude mice. We first assessed whether tumor weight and circulating CD25 level influences blood ^89^Zr-anti-CD25 IgG activity. We then obtained anti-human ^89^Zr-anti-CD25 IgG PET and perform biodistribution studies, and verified tumor CD25-specific targeting of the Ab. We also assessed the distribution of ^89^Zr-anti-CD25 in tumor microsections with autoradiography and histology staining. Furthermore, normal tissue and tumor uptakes of anti-mouse ^89^Zr-anti-CD25 IgG was evaluated in immunocompetent mice bearing lymphomas with low CD25 expression.

## Materials and methods

### Reagents and lymphoma cell culture

Deferoxamine-maleimide was from Macrocyclics (Dallas, TX), and matrigel was purchased from Becton Dickenson Biosciences (Bedford, MA). Tris(2-carboxyethyl)phosphine hydrochloride (TCEP) and phorbol-12-myristate-13-acetate (PMA) was from Sigma-Aldrich (St. Louis, MO). ^89^Zr-oxalate was from the Korea Atomic Energy Research Institute (Daejeon, Korea). Mouse monoclonal IgG2a anti- CD25 Ab (7G7/B6) that is specific against human CD25 and does not react with mouse CD25, rat monoclonal IgG1 Ab against mouse CD25 (PC-61.5.3), and IgG2a isotype control Ab were from BioXcell (West Lebanon, NH). Among antibodies for Western blotting, mouse IgG against β-actin was from Santa Cruz Biotechnology (Dallas, Texas; #sc-47778) and rabbit IgG against CD25 was from Abcam (Cambridge, MA; #ab231441). HRP-conjugated secondary anti-mouse and anti-rabbit IgG was from Cell Signaling Technologies (Beverly, MA).

Human anaplastic large cell lymphoma SUDHL-1 cells and EL4 mouse T lymphoma cells were from the American Type Culture Collection and was tested negative for mycoplasma and authenticated by the Institutional Research Service. Cells were maintained in 5% CO_2_ at 37°C in RPMI 1640 supplemented with 10% fetal bovine serum (FBS), 2 mM L-glutamine, and 100 U/ml penicillin-streptomycin. Cells were sub-cultured twice a week and used when confluence was 80%.

### Preparation of CD25(+) lymphocytes from mouse thymus

The thymus is a vital organ for the development of immune T cells. Normal immune competent C57/BL6 mice were sacrificed by cervical dislocation and the thymus was gently pulled with curved serrated forceps. After surrounding fat and connective tissue was trimmed, the thymus tissue was placed on a 70 μm pore-sized cell strainer (Falcon, NY) and gently mashed through the strainer into a conical tube using the plunger end of a syringe. The homogenate was rinsed with 2% FBS in PBS and centrifuged at 1,200 rpm for 5 min. The supernatant was discarded, and the pellet was resuspended in RBC lysis buffer and incubated at 37°C for 10 min. After adding RPMI-1640 media containing 10% FBS, the cell suspension was centrifuged as above twice to discard cell debris.

After cell were counted using a hemocytometer, CD25(+) and CD25(-) lymphocytes were separated by fluorescence activated cell sorting (FACS). Briefly, 4 × 10^8^ of thymus-isolated cells were incubated with PE-tagged anti-mouse CD25 Ab (Biolegend, #102008, 1:200) in FACS buffer for 30 min at 37°C. FACS was performed on an Aria cell sorter (BD Biosciences, San Diego, CA) using a 488-nm laser excitation channel and a 575-nm fluorescent emission channel. Sorted CD25(-) and CD25(+) lymphocyte cells were seeded and maintained in culture media until experiments.

### Cysteine site-specific deferoxamine conjugation and ^89^Zr labeling

IgG antibodies (2 mg) was reduced at sulfohydryl residues by reaction with 100 mM tris(2-carboxyethyl)phosphine (TCEP) at a 1:100 molar ratio for 20 min at room temperature (RT). The reduced Ab was diluted in 0.1 M sodium phosphate containing 150 mM NaCl and 1 mM EDTA and conjugated with 56.4 µl of 2mM N-(3,11,14,22,25,33-hexaoxo-4,10,15,21,26,32-hexaaza-10,21,32-trihydroxytetratriacontane) maleimide (deferoxamine-maleimide) for 60 min at RT. The molar ratio between deferoxamine-maleimide and Ab was 60:1.

Fifty µl of ^89^Zr-oxalate neutralized with 25 µl of 2 M Na_2_CO_3_ was mixed with deferoxamine-conjugated IgG in 75 µl of 0.5 M HEPES buffer (pH 7.5). Following 60 min incubation at RT, the mixture was loaded on a PD-10 column and eluted with 0.25 M sodium acetate containing 0.5% gentisic acid. Collected 0.5 ml fractions were measured for radioactivity and the peak activity fraction was used.

### Polyacrylamide gel electrophoresis and autoradiography

Ab samples of 2 µg were diluted with water, mixed in 5x non-reducing sample buffer without dithiothreitol, and boiled at 95°C for 10 min. Samples were then separated on an 8% non-reducing sodium dodecyl sulfate (SDS) PAGE by electrophoresis. Protein bands separated on the gel were stained with 0.5% Coomassie blue.

For autoradiography, ^89^Zr- anti-CD25 IgG was loaded without boiling in sample buffer that did not contain SDS or dithiothreitol on an 8% native PAGE and separated by electrophoresis. The gel was dried on a DryEase^®^Mini Cellophane (ThermoFisher Scientific, Waltham, MA) and radioactive bands were detected by X-ray film exposure.

### Radiochemical purity and stability

Radio-instant thin layer chromatography (radio-iTLC) was performed to assess radiochemical purity and stability. ^89^Zr- anti-CD25 IgG was incubated in phosphate buffered saline (PBS) or FBS at 37°C for 0, 1 or 4 days. Radio-iTLC was then performed using 50 mM ethylene diamine tetraacetic acid (EDTA, pH 5.5) as eluent on a glass microfiber chromatography paper impregnated with silica gel. Under these conditions, intact ^89^Zr-anti-CD25 IgG does not change position, whereas free ^89^Zr^4+^ ions and ^89^Zr-EDTA migrate to the solvent front.

### Cell Binding assays and Lindmo assays for immunoreactive fraction

Cell binding assays were performed by incubating cells in RPMI 1640 with 74 kBq of ^89^Zr-CD25 IgG for 60 min at 37°C. Cells were then washed with cold PBS twice, lysed with 0.1 N NaOH, and measured for cell-bound radioactivity. Specificity of cell binding was confirmed by adding 500 nM of unlabeled anti-CD25 IgG prior to ^89^Zr-CD25 IgG incubation.

For Lindmo binding assays, five serial dilutions of SUDHL1 cells (0.1 to 1.6 x 10^7^) in culture medium containing 1% bovine serum albumin (BSA) were incubated with anti-human ^89^Zr-CD25 IgG (final concentration of 50 ng/ml) for 45 min at room temperature (n = 3). The cells were centrifuged and washed twice with ice-cold PBS, and cell-bound radioactivity was counted with a high energy gamma counter. Nonspecific binding was measured in each cell dilutions in the presence of 5 μg/ml of unlabeled CD25 IgG. The immunoreactivity of radiolabeled Ab was estimated as described previously (32). Briefly, a conventional plot was drawn of specific and nonspecific binding over total applied radioactivity, as a function of increasing cell concentration. A double inverse plot was then drawn using the same data as total applied radioactivity over specific binding, as a function of the inverse cell concentration. The immunoreactive fraction was determined through linear extrapolation to the ordinate.

### Western blotting for CD25 protein in cells, blood, and tumor tissue

Immunoblotting methods were previously described (29). Briefly, protein from samples were separated by 10% SDS PAGE and transferred to polyvinylidene fluoride (PVDF) membranes. The membranes were incubated overnight with rabbit primary antibodies against human CD25 (#ab231441; 1:1000) or mouse CD25 (1:1000) at 4°C. The membranes were then washed with TBST buffer and incubated at RT for 1 h with HRP-conjugated secondary anti-rabbit IgG Ab (#7074S; 1:2000 or 1:4000). Immune reactive proteins were detected with an enhanced chemiluminescence system and measured by a GS-800TM calibrated densitometer and Quantity One software (Bio-Rad Laboratories, Hercules, CA). After visualizing target protein, membranes were stripped and re-incubated with antibodies against β-actin (Santa Cruz Biotechnology, sc47778).

### *In vivo* pharmacokinetics, blood activity and tumor uptake of ^89^Zr-anti-CD25 IgG

All animal experiments were conducted in accordance with the National Institute of Health Guide for the Care and Use of Laboratory Animals and were approved by the Institute’s ethics committee. SUHDL-1 tumor models were prepared by subcutaneously injecting cells mixed with 0.1 ml of Matrigel into the right shoulder of 5-week-old male BALB/c nude mice. EL4 tumor models were prepared by subcutaneously injecting cells into the right shoulder of 5-week-old immunocompetent wild type C57/Bl6 mice. Experiments were performed when tumors were palpable 3 weeks after cell inoculation.

For pharmacokinetics studies, 20 SUHDL-1 tumor-bearing mice were intravenously injected with ^89^Zr-anti-CD25 IgG. At 0.5, 17, 48, and 120 h post-injection, five animals were sacrificed by cervical dislocation. The heart was exposed and one hundred μl of blood was collected from the heart using a heparinized syringe. After the blood was centrifuged at 2,000 rpm for 10 min in a heparinized tube, the plasma and pelleted blood cells were transferred to separate tubes and measured for radioactivity on a high energy γ-counter. Whole blood activity was obtained by adding the radioactivity of plasma and blood cells.

In a pilot experiment to test the effects of tumor size on ^89^Zr-anti-CD25 IgG blood activity and tumor uptake, 12 mice were inoculated with SUHDL-1 cells ranging from 5 to 40 ×10^6^ in number that resulted 3 weeks later in tumor weights between 0.1 and 2.0 g. At five days after intravenous injection of ^89^Zr-anti-CD25 IgG, the animals were sacrificed, blood samples and extracted tumors were weighed and measured for radioactivity on a high energy γ-counter. In a separate group of 16 SUHDL-1 tumor-bearing mice, 100 μl of blood collected from the heart at five days after ^89^Zr-anti-CD25 IgG injection and underwent radioactivity measurement Western blotting for assessment of circulating CD25 protein.

### PET and biodistribution in nude mice bearing SUDHL-1 human T lymphomas

Mice bearing SUHDL1 tumors weighing between 0.5 and 1.0 g were randomly allocated into control, Ab blocking, and PMA groups. The latter group was added because PMA is a known inducer of the CD25 gene in T cells, and a pilot *in vitro* test had showed PMA treatment to modestly increase ^89^Zr-CD25 IgG uptake in SUDHL1 cells. This group received intraperitoneal injections with 0.2 mg/kg of PMA (in 5% DMSO) three times over a one-week period. The last PMA injection was three days before sacrifice. The blocking group was intravenously administered with 800 μg of unlabeled anti-human CD25 IgG 1 h prior to ^89^Zr-anti-CD25 IgG injection. A separate control group of tumor mice were injected with ^89^Zr-labeled IgG2a isotype control Ab as a second negative control.

PET imaging was performed five days after tail vein injection of 5.8 MBq anti-human ^89^Zr-labeled Abs. Animals were isoflurane anesthetized and imaged using a Siemens Inveon scanner. The mice were then sacrificed by cervical dislocation, major organs, blood, and tumors were extracted, weighed, and measured for radioactivity.

### PET and biodistribution in wild type mice bearing EL4 mouse T lymphomas

Immunocompetent C57Bl6 mice bearing EL4 mouse T lymphomas weighing between 0.5 and 1.0 g were randomly allocated into control (basal) and blocking groups. Animas were tail vein injected with 4.4 MBq of anti-mouse ^89^Zr-labeled Ab. The blocking group was administered 1 h earlier with 800 μg of unlabeled anti-mouse CD25 IgG. Animals were isoflurane anesthetized five days later and underwent PET imaging and biodistribution studies as above.

### Microsections, autoradiography, and staining of SUDHL1 tumors

SUDHL1 tumors of were extracted from mice at five days after ^89^Zr-CD25 IgG injection. The tissues were snap frozen and cryosectioned at a thickness of 8 μm. The sections were air dried after fixation in 100% acetone at 4°C for 10 min and placed on a photographic film in an X-ray cassette protected from light for adequate durations of exposure. After the films were developed, the autoradiographed images were photographed with a zoom lensed camera. Microscopic sections immediately adjacent underwent alkaline phosphatase staining of microvessels by incubation with substrate solution containing naphthol-AS-MX phosphate free acid (Sigma N4875) and fast red TR hemi salt (Sigma F8764) in 0.1M Tris buffer (pH 8.2) for 15 min at 37°C in a humid chamber. Other adjacent sections underwent immunohistochemistry with a primary anti-human CD25 Ab (Cell signaling #13517; 1:500 dilution) by incubation overnight at 4°C. An EnVision ™ detection system kit (peroxidase-conjugated polymer backbone; DAKO) was used to incubate slides with anti-rabbit secondary Ab. The stained slides were washed, nuclei were lightly counterstained with hematoxylin, and finally mounted with coverslips.

### Statistical analysis

Data are presented as means ± SEM or means ± SD as specified. Differences between groups were analyzed by two-tailed unpaired Student’s t-tests for two groups and ANOVA with Tukey’s *post-hoc* test for three or more groups. Correlations between variables were analyzed by nonparametric Spearman’s analysis with rank correlation coefficients (r). *P* < 0.05 were statistically significant.

## Results

### DFO-conjugation and site-specific CD25 Ab ^89^Zr labeling

**Figure 1:**
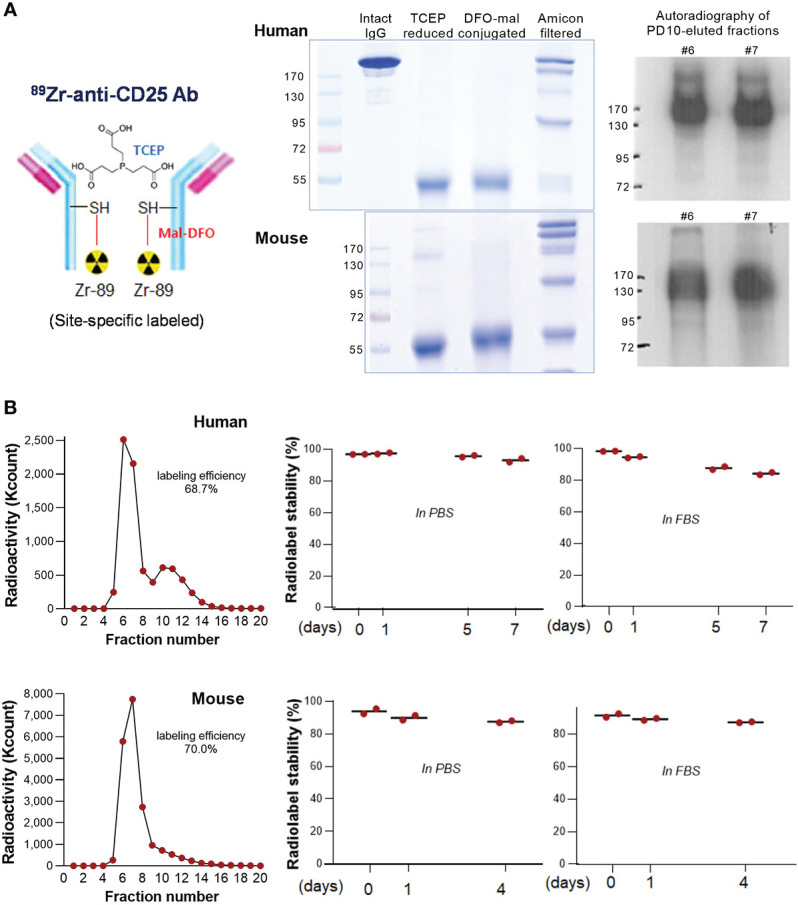
^89^Zr-CD25 IgG characterization. **(A)** Schema of ^89^Zr-CD25 IgG (left). SDS PAGE of intact, TCEP reduced, and DFO-maleimide conjugated anti-human and anti-mouse CD25 IgG (middle). Autoradiography of column-eluted ^89^Zr-CD25 IgG fractions on native PAGE (right). **(B)** PD10 column chromatography of anti-human and anti-mouse ^89^Zr-CD25 IgG (left), and *in vitro* radiochemical stability in PBS and FBS over days (right; mean of duplicate samples per group).

We successfully conjugated monoclonal anti-human CD25 IgG (clone 7G7/B6) and anti-mouse CD25 IgG (clone PC-61.5.3) with ^89^Zr specifically on sulfhydryl residues. Non-reduced SDS-PAGE (**Figure 1A**) showed fragments half the size of the intact Ab after reduction of target disulfide bonds with TCEP. The reduced state was maintained following conjugation with deferoxamine-maleimide (DFO-mal).

^89^Zr radiolabeling of DFO-conjugated anti-human and anti-mouse CD25 IgG was reproducible with labeling efficiencies of 68.7% and 70.0%, respectively (**Figure 1B**). Respective radiochemical purities were 98.3% and 94.0%, and the specific activity was approximately 2.54 mCi/mg. Autoradiography of the first peak elute fractions after PAGE analysis displayed clear radioactive bands at the expected 170 kD region (**Figure 1A**). Radio-iTLC analysis confirmed that radiochemical stability of anti-human CD25 IgG exceeded 93% in PBS and 84% in FBS after 7 days of incubation at 37°C. That of anti- mouse CD25 IgG exceeded 87% in PBS and FBS after 4 days (**Figure 1B**).

### *In vitro* binding of ^89^Zr-CD25 IgG on SUDHL1 lymphoma cells

**Figure 2:**
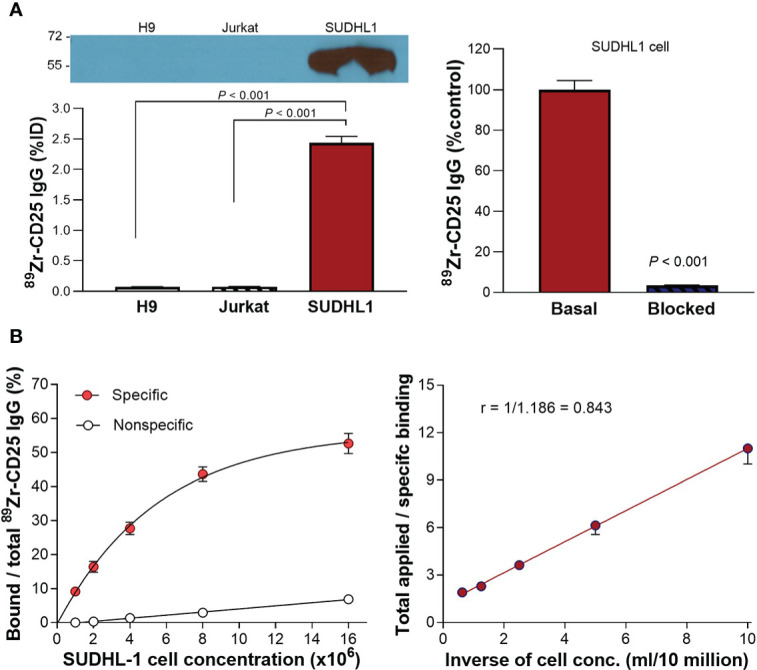
CD25 expression and ^89^Zr-CD25 IgG binding to human lymphoma cells. **(A)** Western blots of CD25 protein and anti-human ^89^Zr-CD25 IgG uptakes in H9, Jurkat, and SUDHL1 lymphoma cells (left). Complete blocking of SUDHL1 cell binding by 0.5 μM of unlabeled anti-CD25 IgG (right). **(B)** Lindmo binding assays for determining the immunoreactive fraction. Five concentrations of SUDHL1 cells were used, from 0.5 to 16 million cells/ml. The final concentration of ^89^Zr-CD25 IgG was 50 ng/ml, and nonspecific binding was assessed with 5 μg of unlabeled anti-CD25 IgG. A conventional plot of specific and nonspecific binding over total applied radioactivity, as a function of increasing cell concentration is shown (left). A double inverse plot was then drawn using the same data as total applied radioactivity over specific binding, as a function of the inverse cell concentration (right). The immunoreactive fraction was determined through linear extrapolation to the ordinate. Data represent the mean ± S.E of values from two independent experiments [total n = 5 per group; uptake in **(A)**], or the mean ± S.D of data from a single representative experiment [n = 3 per group; **(B)**].

Western blot analysis demonstrated that SUDHL1 human T-cell lymphoma cells had high CD25 expression (**Figure 2A**), in contrast to H9 human T-cell lymphoma cells and Jurkat immortalized human T lymphocytes that did not show detectable levels of CD25 protein.

Binding assays demonstrated high anti-human ^89^Zr-CD25 IgG binding to SUDHL1 lymphoma cells that reached 34.2 and 32.0-fold increases over H9 cells and Jurkat cells, respectively (both *P <*0.001; **Figure 2A**). Excellent target-specific binding of ^89^Zr-CD25 IgG confirmed by blocking with 0.5 μM of unlabeled anti-CD25 IgG that abolished the high SUDHL1 cell uptake at baseline to 3.3 ± 0.2% (*P <*0.001; **Figure 2A**).

Based on immunoreactivity assays using SUDHL-1 cells and the double inverse plot method by Lindmo et al., the immunoreactive fraction of anti-human ^89^Zr-CD25 IgG was calculated as 84.3% (**Figure 2B**). However, the conventional plot of specific binding over total applied radioactivity, as a function of increasing cell concentration displayed a lower apparent Bmax. It should therefore be noted that the true immunoreactive fraction may be somewhat lower, and perhaps closer to 60%.

### Influence of tumor size and circulating CD25 on blood ^89^Zr-CD25 IgG activity

**Figure 3:**
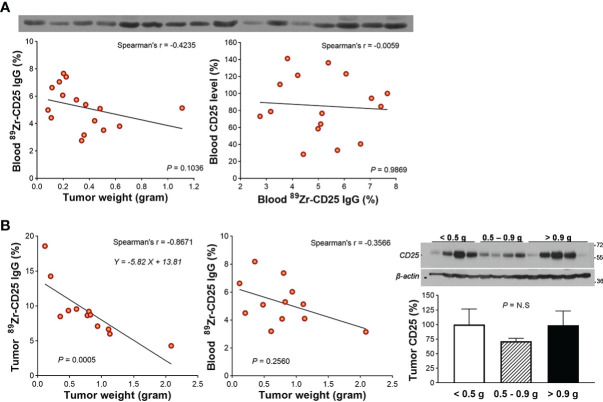
Circulating free CD25 level and effect on blood ^89^Zr-CD25 IgG. **(A)** Western blots of CD25 protein in blood obtained from the heart of 16 SUDHL1 lymphoma-bearing mice (top). Relations of tumor weight with blood ^89^Zr-CD25 IgG (bottom left) and blood ^89^Zr-CD25 IgG with blood human CD25 level (bottom right). **(B)** Relations of tumor weight with ^89^Zr-CD25 IgG tumor uptake (left) and blood activity (middle) in another group of mice. Western blots and quantified band intensities of tumor CD25 protein according to tumor weight group is shown in the right. Lines were fitted by linear regression and correlation was based on nonparametric Spearman’s analysis. Bars represent the mean ± S.D of four samples per group. N.S., not significant.

We first piloted whether large variations in tumor size or level of circulating CD25 protein released from the tumor might influence blood activity of ^89^Zr-CD25 IgG. The results showed that divergent tumor weights between 0.08 and 0.63 g did not influence blood ^89^Zr-CD25 IgG level at day five (Spearman’s r = -0.4235, *P* = 0.103; **Figure 3A**). Moreover, Western blots of protein from the blood of the mice revealed no correlation between blood ^89^Zr-CD25 IgG activity and level of human CD25 protein in the blood (Spearman’s r = -0.0059, *P* = 0.987; **Figure 3A**).

A second group of mice showed that large SUDHL1 tumors were correlated with lower average ^89^Zr-CD25 IgG uptake per tumor weight (Spearman’s r = -0.867, *P* = 0.0005; **Figure 3B**), but again, blood ^89^Zr-CD25 IgG activity was not significantly influenced (Spearman’s r = -0.357, *P* = 0.2560; **Figure 3B**). Western blots of the tumor tissue showed that CD25 protein level was not different between small (0.10-0.45 g; relative CD25 band density, 100.0 ± 26.4%), moderate (0.45-0.93 g; 71.7 ± 9.4%), and large tumors (1.00-3.00 g; 98.7 ± 54.7%; **Figure 3B**).

### *In vivo* pharmacokinetics of ^89^Zr-anti-CD25 IgG in the plasma and blood cells

**Figure 4:**
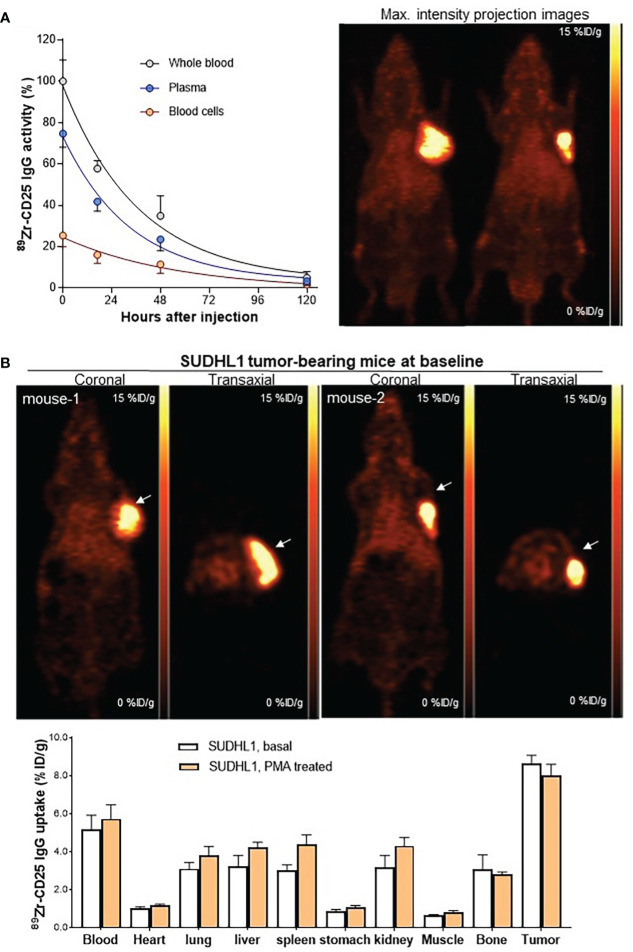
Pharmacokinetics, PET imaging, and biodistribution in mice. **(A)** Anti-human ^89^Zr-CD25 IgG activity in whole blood, plasma, and blood cells of SUDHL1 lymphoma mice following intravenous injection (left). Data are the mean ± SD of five mice for each time group. Representative maximum intensity projection PET images at 5 days (right). **(B)** Representative coronal (top left) and transaxial (top right) PET images. Biodistribution at 5 days in mice at baseline or after three intraperitoneal PMA injections. Data are the mean ± S.E from two independent experiments (n = 5 per group).

The remaining animal studies including pharmacokinetics, PET, and biodistribution studies were performed in mice with SUDHL1 tumors between 0.5 to 1.0 g. *In vivo* pharmacokinetic analysis in these animals demonstrated exponential reductions of plasma, blood cell, and whole blood activity of intravenously injected anti-human ^89^Zr-anti-CD25 IgG (**Figure 4A**). In the whole blood, ^89^Zr-anti-CD25 IgG activity decreased to 57.7± 3.7% and 34.9± 9.7% of the initial 30 min level at 17 h and 48 h, respectively. The K value was 0.027 and the calculated whole blood half-life was 26.0 h (95% confidence interval, 17.6 to 40.8 h). ^89^Zr-anti-CD25 IgG activity in the plasma represented three fourths to two thirds (66.2 to 72.3%) of whole blood activity throughout five days. Pharmacokinetics of plasma activity closely followed that of whole blood, with a K value of 0.029 and the calculated half-life of 23.3 h (95% confidence interval, 16.6 to 33.5 h). In comparison, blood cells initially took up only 25.3% of whole blood activity but had a longer half-life of 39.2 h.

### PET imaging and biodistribution in nude mice bearing SUDHL-1 human T lymphomas

Anti-human ^89^Zr-CD25 IgG PET in mice at five days post-injection displayed clear SUDHL1 lymphoma visualization with low uptake in normal organs (**Figures 4A, B**). The biodistribution studies that followed confirmed high tumor uptake of ^89^Zr-CD25 IgG at 8.7 ± 0.9%ID/g (**Figure 4B**). This was followed by blood activity at 5.2 ± 1.6%ID/g, and lower uptakes in the heart, lung, liver, spleen, and kidneys between 1.0 and 3.5%ID/g. Muscle uptake was remarkably low at 0.7 ± 0.0%ID/g (**Figure 4B**).

In a separate group of mice treated with intraperitoneal PMA injection, biodistribution results showed no change of tumor ^89^Zr-CD25 IgG uptake (8.0 ± 1.4%ID/g) or blood activity (5.7 ± 1.7%ID/g), compared to those of untreated controls (both *P* = NS; **Figure 4B**).

**Figure 5:**
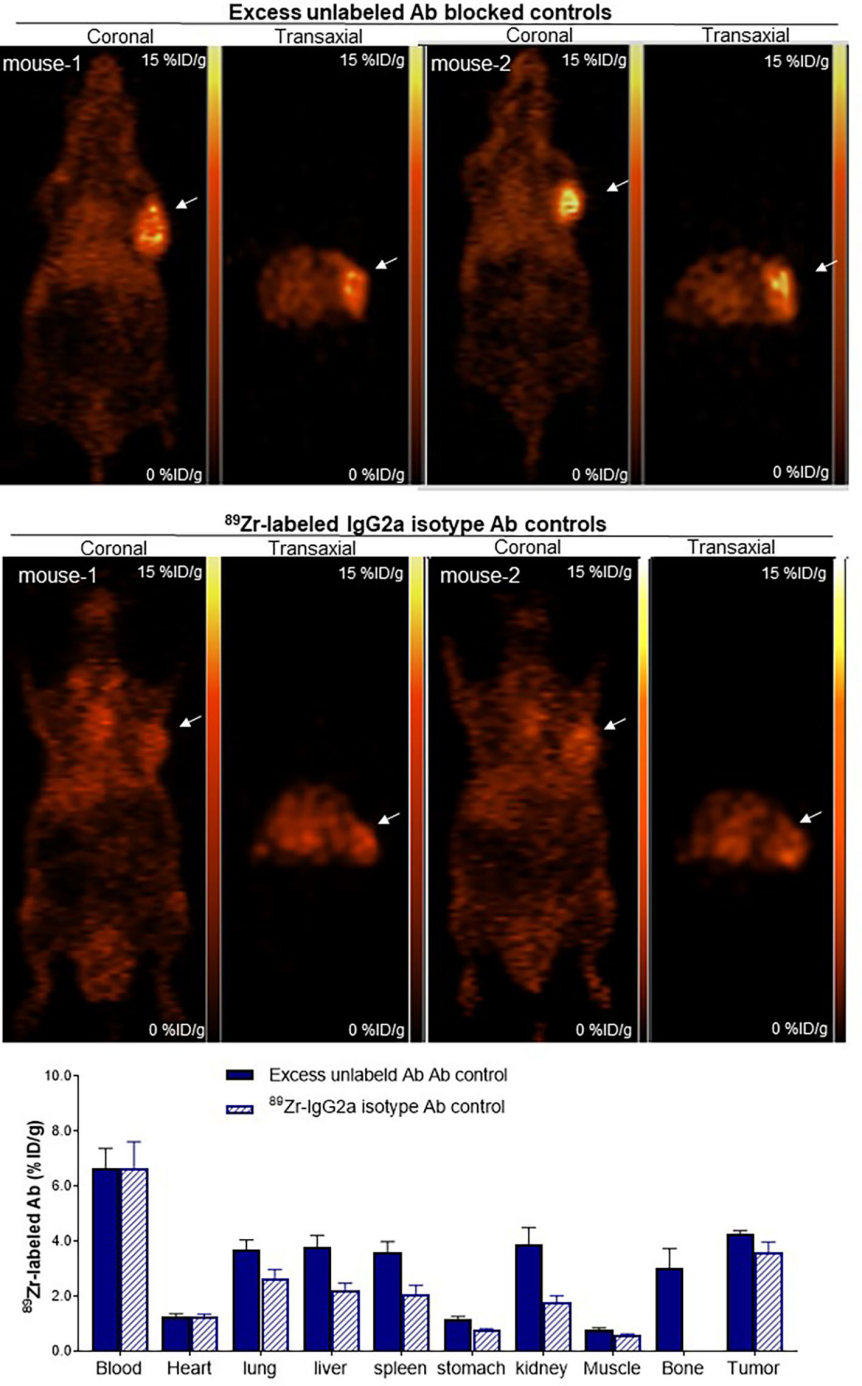
Target Specificity of tumor uptake *in vivo*. Representative coronal (left) and transaxial (right) PET images at 5 days of SUDHL1 lymphoma mice co-injected with anti-human ^89^Zr-CD25 IgG and 0.8 mg of unlabeled anti-CD25 IgG for blocking (top), or with ^89^Zr-labeled IgG2a isotype control Ab (middle). Biodistribution data are shown (bottom) as the mean ± S.E of values obtained from two independent experiments (n = 5 per group).

When animals were co-injected with excess unlabeled anti-CD25 IgG, tumor uptake was substantially reduced (**Figure 5**). Biodistribution studies showed that Ab blocking reduced tumor uptake to 4.3 ± 0.2%ID/g (50.4% suppression compared to basal level), demonstrating specific tumor targeting of ^89^Zr-CD25 IgG *in vivo* (**Figure 5**). Blood activity (6.7 ± 1.6%ID/g) and uptake in normal organs were not influenced by unlabeled anti-CD25 IgG administration.

Another control group of SUDHL1 tumor mice injected with ^89^Zr-labeled IgG2a isotype control Ab displayed even lower tumor uptake (**Figure 5**), further supporting that ^89^Zr-CD25 IgG offers target specific tumor uptake. These mice showed activities in the blood (6.7 ± 2.3%ID/g) and normal organs (between 1.0 and 2.6%ID/g) that was similar to those of ^89^Zr-CD25 IgG. However, tumor uptake of ^89^Zr-labeled IgG2a isotype control Ab was low at 3.6 ± 0.9%ID, which was even lower than tumor ^89^Zr-CD25 IgG uptake in the presence of excess unlabeled anti-CD25 IgG (**Figure 5**).

### Mouse cells and PET imaging and biodistribution in EL4 tumor of wild type mice

**Figure 6:**
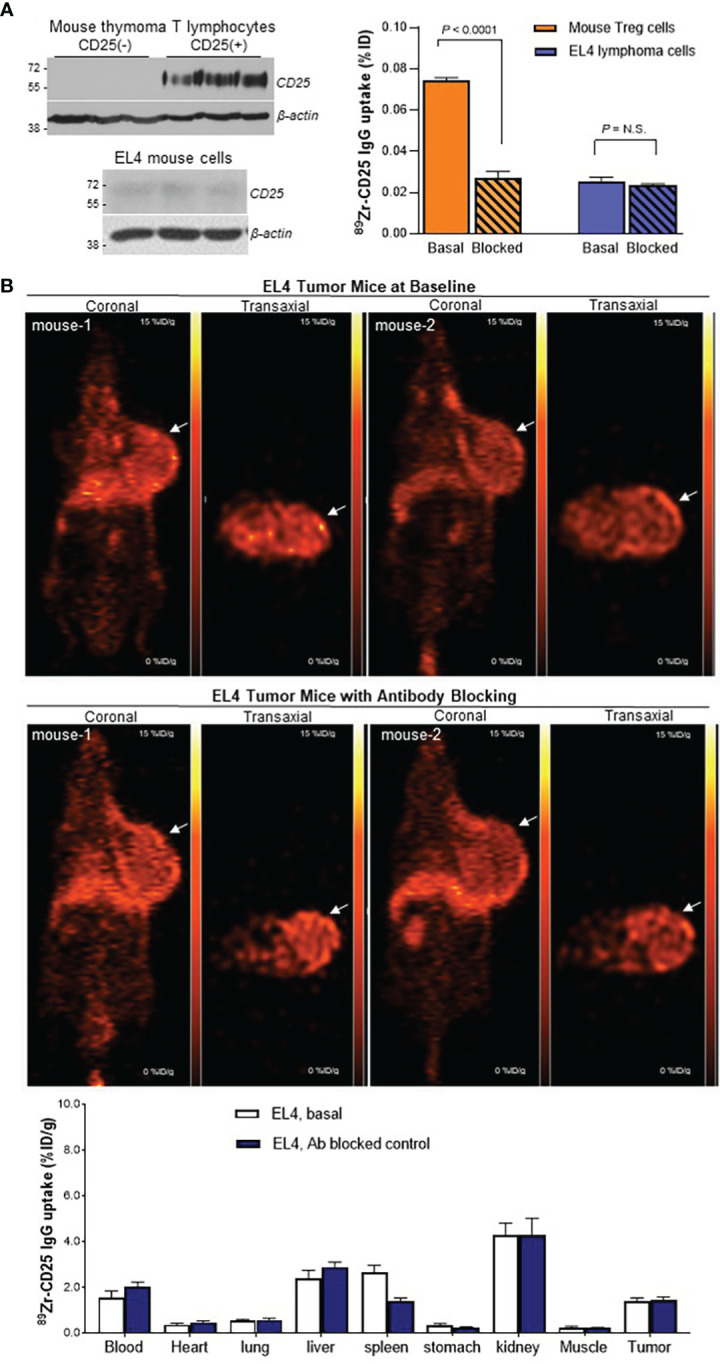
^89^Zr-CD25 IgG uptake in mouse cells *in vitro* and in mouse lymphoma *in vivo*. **(A)** CD25 expression (left) and anti-mouse ^89^Zr-CD25 IgG binding (right) in mouse thymus Treg lymphocytes and EL4 mouse lymphoma cells. Cell uptake was compared by using equivalent amounts of cells. Blocking was done by adding 0.5 μM of unlabeled anti-CD25 IgG. **(B)** Representative coronal (left) and transaxial (right) PET images at 5 days of EL4 tumor-bearing immunocompetent C57/Bl6 mice administered with anti-mouse ^89^Zr-CD25 IgG alone (top) or co-injected and 0.8 mg of unlabeled anti-CD25 IgG for blocking (middle). Biodistribution data are shown (bottom) as the mean ± S.E of values obtained from two independent experiments (n = 6 per group) N.S., not significant.

FACS of mouse thymus-derived lymphocytes separated CD25(+) Tregs and CD25(-) lymphocytes, which showed high and low CD25 expression, respectively (**Figure 6A**). CD25(+) lymphocytes displayed high anti-mouse ^89^Zr-CD25 IgG uptake that reached 4-fold increases over CD25(-) lymphocytes (*P <*0.001), which was suppressed to CD25(-) cell level in the presence of 0.5 μM of unlabeled anti-CD25 IgG (*P <*0.001; **Figure 6A**). In comparison, EL4 mouse T lymphoma cells showed very low CD25 expression and very low levels of specific anti-mouse ^89^Zr-CD25 IgG uptake (**Figure 6A**). EL4 cells were thus used for a syngeneic tumor model to test anti-mouse ^89^Zr-CD25 IgG distribution in immunocompetent animals.

When immunocompetent mice bearing EL4 mouse lymphomas were injected with anti-mouse ^89^Zr-CD25 IgG, PET images at five days displayed low uptake in normal organs as well as in the lymphoma tumor (**Figure 6B**). Biodistribution data showed that uptake in the tumor with low CD25 expression was only 1.4 ± 0.4%ID/g, which is lower than Ab blocked ^89^Zr-CD25 IgG uptake and ^89^Zr-labeled IgG2a isotype IgG uptake in SUDHL1 tumors. Blocking with excess unlabeled anti-CD25 IgG did not further decrease tumor uptake that was already low (1.4 ± 0.3%ID/g; **Figure 6B**), indicating absence of significant amounts of specific uptake in the tumor.

Importantly, ^89^Zr-CD25 IgG uptake in these immunocompetent mice did not demonstrate increased uptake in the blood (2.0 ± 0.5%ID/g), spleen (1.4 ± 0.4%ID/g), or liver (2.9 ± 0.5%ID/g), compared to nude mice. Again, Ab blocking did not further decrease uptake in these organs.

### Microsections, autoradiography, and staining of SUDHL1 tumors

**Figure 7:**
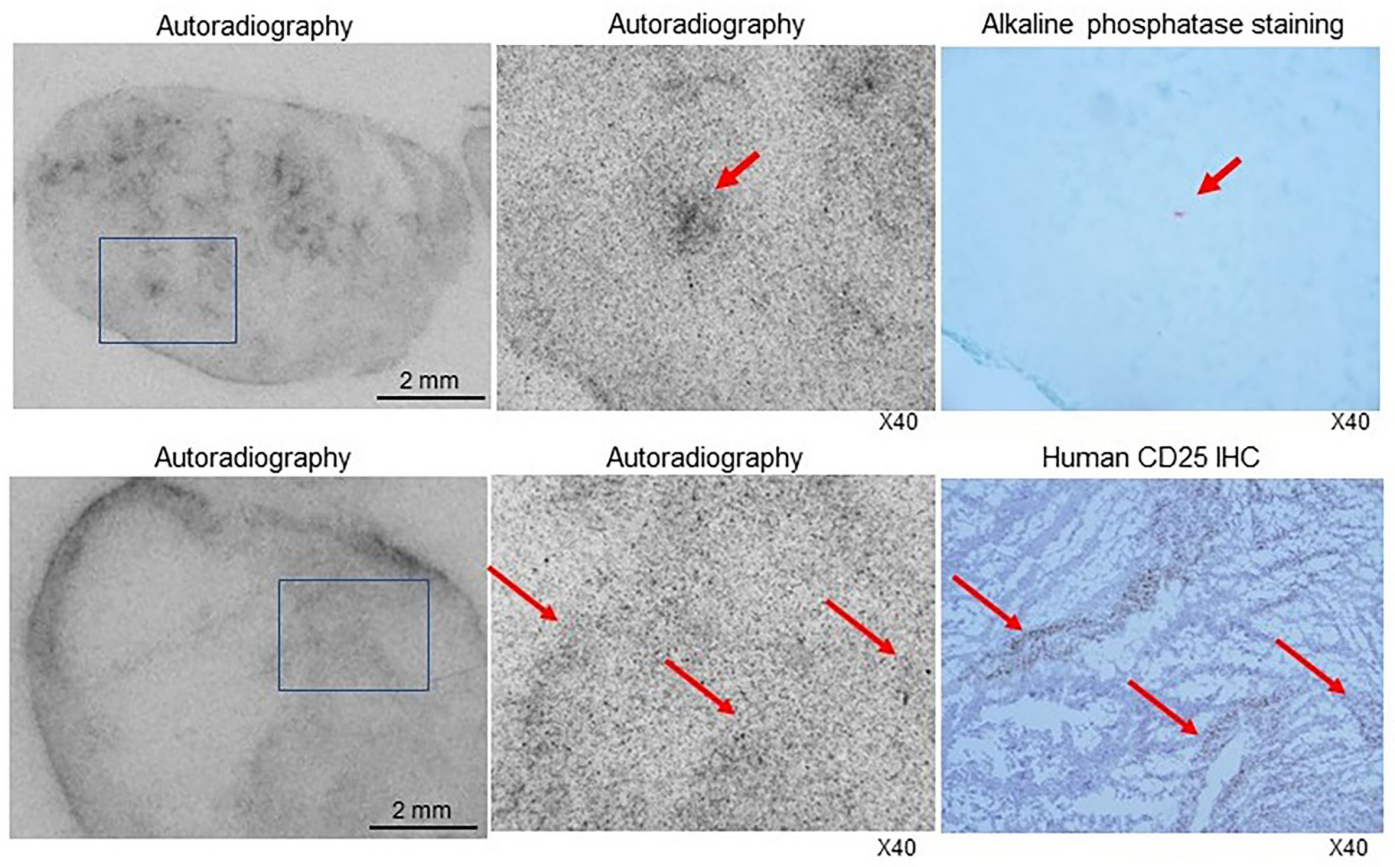
Autoradiography and histologic staining of microsections of SUDHL1 tumors from ^89^Zr-CD25 IgG- injected mice. (Top) Photograph of a film exposed to a microsection of a small tumor showing radioactivity efficiently delivered into the inner tumor regions (left). A microscopic image of the tumor region in the boxed area demonstrates a focus of greater radioactivity (middle) that contained microvessels shown in red by alkaline phosphatase staining in an immediately adjacent microsection (right). (Bottom) Photograph of a film exposed to a microsection of a larger tumor showing greater radiation accumulated in the tumor periphery (left). A microscopic image of the tumor region in the boxed area demonstrates radioactivity distribution (middle) that correlated with immunohistochemistry staining for human CD25 (right).

The autoradiography and histology findings of SUDHL1 tumor microsections demonstrated that ^89^Zr-CD25 IgG distribution in smaller tumors was rather uniform with efficient delivery into inner tumor areas (**Figure 7**). There was also a tendency for greater tracer accumulation near microvessels. In contrast, larger tumors showed greater accumulation in tumor peripheral, adjacent to vessel rich adventitial tissues. Yet, radioactivity accumulation inside the tumor demonstrated correlation with CD25 expression (**Figure 7**).

## Discussion

IL-2Rα is highly expressed in hematologic malignancies and mediates oncogenic signaling. Therefore, Ab therapeutics against this receptor offer a unique opportunity for treating refractory lymphomas. Anti‐CD25 Abs conjugated with toxic agents or therapeutic radioisotopes are being developed, with some undergoing early trials to establish clinical safety and efficacy. In this study, we developed a site-specific ^89^Zr-based immuno-PET technique that provides noninvasive imaging of CD25 status on T cell lymphomas *in vivo*.

For this purpose, we used 7G7/B6, a mouse monoclonal IgG2a that specifically targets and binds to human CD25 at an extracellular domain different from that targeted by daclizumab. 7G7/B6 Ab has previously been used to investigate the structure-function relationship of IL-2R (33). It has also been used as a therapeutic Ab (34) and as radioimmunoconjugates against CD25(+) lymphomas (10–12). In our study, 7G7/B6 was labeled with ^89^Zr by a cysteine residue-specific conjugation method using DFO-maleimide for favorable radioprobe homogeneity and immuno-reactivity. This elegant way of tailoring the ^89^Zr attachment site on Abs has not been previously used for anti-CD25 Abs. Our group has shown that mild reaction with TCEP induces cysteine site-specific reduction of Abs for DFO conjugation, which likely occurs at the two hinge region disulfide bonds (29). We have successfully used this straightforward site-specific ^89^Zr labeling method for immuno-PET of tumor CD44 (27), CD133 (28), programmed cell death ligand-1 (29), and programmed cell death-1 (30).

The CD25 receptor is a promising theranostic target because its expression is largely limited to malignant lymphomas and selected types regulatory and activated T lymphocytes. Most adult T cell leukemia/lymphoma and hairy cell leukemia cells constitutively express CD25, and peripheral T cell lymphomas including a particularly aggressive type called anaplastic large cell lymphoma have characteristically high CD25 expression (35). Our Western blots demonstrated strong CD25 bands on human anaplastic large cell lymphoma SUDHL1 cells. These cells revealed high ^89^Zr-CD25 IgG uptake that exceeded 30-fold of that by CD25(-) human lymphoma cells. Furthermore, excellent target specificity of binding was confirmed by complete loss of ^89^Zr-CD25 IgG uptake by SUDHL1 cells in the presence of excess unlabeled CD25 IgG.

Using anti-mouse ^89^Zr-CD25 IgG, we showed that mouse thymus-derived lymphocytes selected for positive CD25 expression have high ^89^Zr-CD25 IgG uptake compared to CD25(-) lymphocytes. Again, this uptake was substantially suppressed by excess unlabeled CD25 IgG.

*In vivo* pharmacokinetic studies in SUHDL-1 tumor-bearing mice demonstrated that after intravenous injection of ^89^Zr-anti-CD25 IgG, plasma activity represented three fourths to two thirds of whole blood activity with highly similar exponential clearance rates and half-lives. In comparison, blood cells took up a much smaller fraction of circulating activity, indicating that blood ^89^Zr-anti-CD25 IgG resides predominantly in the plasma space.

Before initiating full-scale animal experiments, we first conducted pilot tests to assess the influence of tumor size on ^89^Zr-CD25 IgG activity in tumor and blood. This was prompted by a previous study that observed reduced uptake of radiolabeled anti-CD25 Ab fragments in larger CD25(+) tumors. The authors suggested that this could be attributed to greater release of soluble IL2-Rα that took up the radiotracer (23). Our pilot test also showed an inverse relationship between tumor weight and ^89^Zr-CD25 IgG uptake. Mechanistically, however, this did not appear to be caused by greater amounts of circulating CD25 protein. Firstly, blood ^89^Zr-CD25 IgG activity was not influenced by tumor weight. Secondly, serum CD25 protein in a separate group of mice was similar across mice with different tumor weights. Lastly, blocking experiments showed that blood ^89^Zr-CD25 IgG activity was not reduced but appeared slightly increased in the presence of excess unlabeled Ab. We therefore suggest that reduced Ab penetration into the core of larger tumors is more likely responsible for the observed lower uptake. Indeed, our autoradiography and histology findings of tumor microsections showed that unlike smaller tumors that had rather uniform radiotracer distribution extending into inner tumor regions, uptake in larger tumors tended to be greater in tumor periphery, where vascularization was more abundant. Therefore, the inability of larger tumors to proportionately increase uptake could be due to limited Ab delivery to their tumor core.

In murine models with SUDHL1 lymphomas of appropriate size between 0.5 and 1.0 g, ^89^Zr-CD25 IgG PET displayed clear tumor visualization with excellent contrast and showed low uptake in normal organs. Biodistribution studies reiterated the PET findings by demonstrating high tumor uptake and significantly lower uptake in other organs. Radioactivity in the bones was also low, indicating low amounts of free ^89^Zr that is known to accumulate in bones (36).

In our results, CD25 protein expression seemed to vary even within groups of different tumor size. External factors such as perfusion and oxygenation status, proliferation, apoptosis or necrosis, can obviously exert large influences on the expression status of cell surface markers. However, it may be possible that CD25 expression on lymphoma cells can also be regulated by specific signaling pathways, similar to that of immune T cells. It should therefore be interesting to investigate specific signaling pathways that may modulate CD25 expression in lymphomas *in vivo*. Protein kinase C activation has an established role in the molecular control of CD25 expression in normal T cells (37). Furthermore, treatment of Jurkat T lymphoblasts with the protein kinase C activator PMA has been shown to upregulate CD25 (38). We therefore tested the effect of repeated PMA administration but found that it failed to increase ^89^Zr-CD25 IgG uptake in SUDHL1 tumors. This might indicate attenuation of protein kinase C-mediated CD25 regulation in lymphoma cells, but this will require further investigations for clarification.

When animals were pre-injected with excess unlabeled anti-CD25 Ab, tumor ^89^Zr-CD25 IgG uptake was blocked on PET images as well as on biodistribution measurements. In addition, there was low tumor uptake of ^89^Zr-labeled IgG2a isotype control Ab. Together, these results confirm high target specificity *in vivo*. As mentioned, blocking of tumor uptake with unlabeled Ab was not associated with reduced blood ^89^Zr-CD25 IgG activity, indicating that binding to circulating CD25 protein was minor.

The ^89^Zr-CD25 IgG we administered contained 10 μg of Ab, which is well below the 100 ug of 7G7/B6 anti-CD25 Ab that was previously shown to inhibit leukemia cell growth in mice (10). Similar tumor sizes at day five between mice injected with ^89^Zr-CD25 IgG and those injected with ^89^Zr-isotype control Ab (789 ± 118 mg vs. 791 ± 262 mg) in our study further supports the lack of any therapeutic efficacy of ^89^Zr-CD25 IgG that might limit its applicability in the clinics.

As mentioned, regulatory and activated T cells also express CD25 and our results also confirm specific ^89^Zr-CD25 IgG uptake in mouse regulatory T cells. This could potentially influence ^89^Zr-CD25 IgG biodistribution and PET imaging *in vivo*. The SUDLH1 tumor model circumvented this factor by using anti-human Abs and immune-deficient mice. Indeed, the 7G7/B6 Ab used for ^89^Zr-CD25 IgG is specific against human CD25 and does not bind murine T cells. We therefore also included experiments using anti-mouse ^89^Zr-CD25 IgG in immunocompetent mice bearing EL4 mouse lymphomas that have very low CD25 expression. The results showing low uptake in the tumors as well as in normal organs including liver and spleen, and the inability of excess unlabeled Ab to further reduce the already low uptake, points to minimal specific ^89^Zr-CD25 IgG uptake in the tissues. This finding provides support that CD25+ T cells does not significantly influence ^89^Zr-CD25 IgG distribution and PET imaging of lymphomas.

Nonetheless, a limitation of the present study is that we did not directly test whether ^89^Zr anti-CD25 IgG PET could image tumor infiltrating CD25(+) T cells *in vivo*. Further investigations are thus required to clarify the clinical relevance of CD25-targeted PET for imaging of tumor infiltrating T cells by administration of T cells to tumor models in a separate set of well-designed experiments.

Finally, newer generation Ab therapeutics are in development, including Ab fragments such as single domain antibodies, nanobodies, and Ab mimetics such as affibodies. Since these Ab derivatives have faster *in vivo* kinetics compared to intact Ab, future investigations could demonstrate whether ^89^Zr conjugation of these formats allows PET imaging of tumor CD25 at earlier time points.

In conclusion, ^89^Zr-CD25 IgG prepared by cysteine site-specific labeling targeted human SUDHL1 T lymphoma cells with high affinity and specificity. In immune-deficient mice bearing SUDHL1 lymphomas, ^89^Zr-CD25 IgG PET provided high-contrast tumor imaging, and CD25-specific tumor targeting was confirmed. ^89^Zr-CD25 IgG immuno-PET may thus be useful for imaging of T-cell lymphomas and noninvasive assessment of CD25 expression on target cells *in vivo*.

## Data availability statement

The original contributions presented in the study are included in the article/supplementary material. Further inquiries can be directed to the corresponding author.

## Ethics statement

The animal study was reviewed and approved by the Institute Ethics Committee of Samsung Medical Center.

## Author contributions

K-HL conceived of the theoretical basis for the implementation of immune-PET imaging of CD25. *In vivo* work was performed by JL, K-HJ, and MK. Radiotracer labeling was performed by JL and K-HJ. Imaging analysis was performed by K-HL and K-HJ. *Ex vivo* work was performed by JL and MK. Manuscript was written by K-HL, JL and K-HJ. JL and K-HJ contributed equally to this work. All authors contributed to the article and approved the submitted version
